# Automated silylation of flavonoids using 3D printed microfluidics prior to chromatographic analysis: system development

**DOI:** 10.1007/s00216-023-04981-4

**Published:** 2023-10-07

**Authors:** Thabang Bernette Ncongwane, Derek Tantoh Ndinteh, Elize Smit

**Affiliations:** Center for Natural Products Research, Department of Chemical Sciences, University of Johannesburg, Auckland Park, PO Box 524, Johannesburg, South Africa

**Keywords:** Flavonoids, Derivatization, Microfluidics, 3D printing, Automation

## Abstract

Flavonoids are a class of secondary plant metabolites with low molecular weights. Most flavonoids are highly polar and unsuitable for gas chromatographic analyses. Derivatization is commonly used to make them amenable to gas chromatography by altering their physicochemical properties. Although highly effective, derivatization techniques introduce extra preparation steps and often use hazardous chemicals. The aim of this study was to automate derivatization (specifically, silylation) by developing 3D printed microfluidic devices in which derivatization of flavonoids can occur. A microfluidic device was designed and 3D printed using clear polypropylene. Quercetin and other flavonoids (TED 13 and ZTF 1016) isolated from plant extracts were silylated with N-tert-butyldimethylsilyl-N-methyltrifluoroacetamide (MTBSTFA) at room temperature both in batch and in continuous flow. All the samples were analyzed using Fourier transform infrared (FTIR) spectroscopy, gas chromatography combined with mass spectrometry (GC–MS), and high-resolution accurate mass spectrometry (HR-MS). Interestingly, the HR-MS results showed that the flow method was about 25 times more efficient than the batch method for quercetin samples. The TED 13 flavonoid was completely derivatized in the flow method compared to the batch method where the reaction was incomplete. Similar results were observed for ZTF 1016, where the flow method resulted in a four times derivatized compound, while the compound was only derivatized once in batch. In conclusion, 3D printed microfluidic devices have been developed and used to demonstrate a semi-automated, inexpensive, and more efficient natural product derivatization method based on continuous flow chemistry as an alternative to the traditional batch method.

**Supplementary Information:**

The online version contains supplementary material available at 10.1007/s00216-023-04981-4.

## Introduction

Flavonoids are a group of naturally occurring polyphenolic compounds, a class of secondary plant metabolites with low molecular weights [1]. Plants, such as fruits and vegetables, use flavonoids for growth and defense against pathogens. Hence, they contain larger quantities of flavonoids [2]. According to Panche et al., these compounds can be identified as pigments in most flowering plants, although they are also found throughout other parts of the plant [3]. These flavonoids are characterized by diverse chemical structures and are often extracted as complex mixtures. A systematic review has shown that the prenylated derivates show improved biological activities, e.g., antioxidant, antibacterial, antiviral, anti-inflammatory, anticancer, and hepatoprotective activity [1, 4].

Flavonoids can be analyzed using various techniques, with high-performance liquid chromatography (HPLC) and gas chromatography (GC) being used most often [5–9]. Due to the complexity of the sample extracts/mixtures, chromatographic separations are often combined with mass spectrometric detection, which provides qualitative information [10]. The advantages and disadvantages of using HPLC and GC for flavonoid analysis are summarized in Table 1.

**Table 1:** Properties of HPLC and GC that indicate their advantages and disadvantages in terms of flavonoid analysis

Property	HPLC [7, 10–13]	GC [6, 8, 12, 14]
Stationary phase	Solid	Liquid
Mobile phase	Liquid	Gas
Analysis time	Longer	Shorter
Separation efficiency	Lower	Higher
Selectivity	Higher	Lower
Application range	Separation of a wide spectrum of compounds	Limited to semi-volatile, volatile, and thermally stable compounds
Sample preparation	Minimal	More sample preparation required (e.g., derivatization)
Peak capacity	Lower	Higher
Resolution	High^a^	Low^a^

^a^Resolving power of complex samples with similar retention times

Most flavonoids are highly polar, have low volatility, and have limited thermal stability. Although GC is a simpler, more cost-effective, and environmentally friendly technique compared to HPLC, the limited range of analytes (i.e., volatile, semi-volatile, and thermally stable compounds) amenable to GC analysis has hindered its widespread implementation for flavonoid analysis. This is clearly evident when looking at the number of publications reported on HPLC when compared to GC analysis of flavonoids [6, 11].

However, through derivatization, the range of compounds that can be analyzed with GC can be expanded significantly, by improving their volatility and thermal stability [12]. The first work on GC analysis of derivatized flavonoids was reported in 1962 [13]. In their work, flavonoids were derivatized using methylation (with dimethyl sulphate) and subsequently analyzed using GC combined with thermal conductivity detection. Although the work was reported more than six decades ago, it has only recently received much attention. This is attributed to the development of high-temperature GC (HTGC) [14], and improved derivatization techniques [15]. Some newer developments include multidimensional GC, a technique that has also recently enjoyed wide attention in the analysis of minor flavonoids which is commonly difficult with HPLC [6, 16, 17].

Fiamegos et al. used GC–MS to characterize derivatized flavonoids and phenolic acids from plant extracts. The derivatization (methylation) and chromatographic separation took 45 min and good separation was reported [11]. Another GC–MS technique was developed by Fernandez et al. for identification of compounds from wood extract of *Populus tremuloides*. Interestingly, all 70 target compounds were easily identified in the underivatized sample compared to the tert-butyldimethylsilyl chloride (TBDMS) derivatized sample, and they concluded that derivatization was unnecessary when using GC–MS for analysis of volatile/semi-volatile target compounds [18]. Quercetin together with catechin and resveratrol isolated from biological fluids (blood, serum, and urine) has been derivatized using BSTFA (N,O-bis(trimethylsilyl) trifluoroacetamide). The analysis was also performed using GC–MS, and they concluded that derivatization is highly recommended for analysis of these flavonoids in biological fluids due to their improved resolution and sensitivity [19]. In another study, flavonoids and phenolic acids were extracted from human plasma and subsequently derivatized using BSTFA + TMCS (trimethylchlorosilane); an average recovery of 79.3% was observed, also using GC–MS [5]. A few other studies on GC–MS analysis of derivatized flavonoids have been reported [20–22].

Although highly effective, derivatization techniques are often expensive, are laborious, and introduce extra sample preparation steps [23]. These experiments also require the use of hazardous chemicals and is often performed in batch under harsh conditions that require controlled environments (e.g., an inert atmosphere) [24]. Importantly, imperfectly controlled chemical reactions can also increase uncertainty in analytical results [12]. Automation of derivatization steps is needed to address these limitations. While high-end autosamplers have shown some potential, a more economic approach is required to expand the application of GC in flavonoid analysis.

Flow chemistry provides an alternative to traditional (and manual) batch reactions [25, 26]. Also referred to as continuous flow chemistry, it is characterized by the use of channels or tubing to perform chemical reactions in a continuous stream instead of a flask [25–27]. Performing reactions in flow using microfluidics offers benefits such as low cost, reduction of human error margins, rapid diffusional mixing due to laminar flow and high surface area to volume ratio, efficient heat/mass transfer rates, and faster and safe reactions in case of hazardous chemicals. It also provides high selectivity due to low variations in temperature, concentration and addition rates [26, 28].

Microfluidics have been applied to the study of natural products with a focus on active compounds and drug discovery, as discussed in a recent review [29]. However, to the best of our knowledge, microfluidics has not been applied to derivatization of natural products to make them amenable to GC analysis. Based on the above-mentioned advantages of microfluidics, it is ideal for automation of derivatization procedures, which is crucial to improve throughput, selectivity, and capacity in natural product analysis.

Microfluidic devices are commonly fabricated using numerous techniques that are complex, specialized, expensive, and often time-consuming (e.g., etching of glass). Alternatively, 3D printing (e.g., fused deposition modeling, FDM) can be adopted as an inexpensive rapid prototyping process of creating an object by stacking material in a layer-by-layer sequence [30]. FDM is extrusion-based and has shown the potential to manufacture high-quality microfluidic devices [28]. The benefits of 3D printed devices include rapid prototyping, customizability, reusability, low cost, and not requiring specialized skills [30, 31]. These can then be combined with open-source pump-based flow equipment to build a low-cost flow system [28, 32]. Commercial flow equipment is available but expensive and specialized.

The aim of this research was to develop a 3D printed flow system to perform derivatization, specifically silylation, in an automated way. The end goal is to streamline derivatization of flavonoids and consequently promote the use of GC in natural product analysis. In the work presented here, quercetin was used as a model compound, followed by other flavonoids extracted from plants. Derivatization was performed in flow and in batch to evaluate and compare the efficiency of the derivatization process. Various analytical tools, including GC, Fourier transform infrared (FTIR) spectroscopy, and high-resolution mass spectrometry (HR-MS), were used to prove that derivatization took place, thus validating the developed system.

## Materials and methods

### Chemicals and materials

**Fig. 1:**
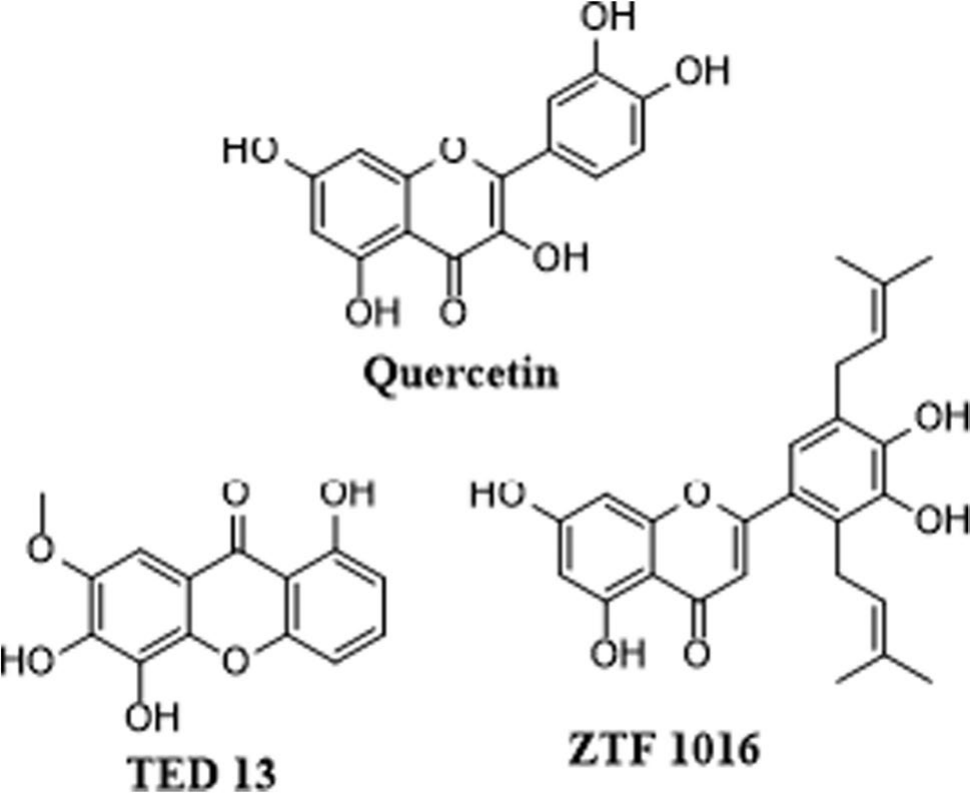
Structures of flavonoids used in this study include quercetin, TED 13, and ZTF 1016. TED 13 was isolated from plant extracts [33, 34]. ZTF 1016 was recently isolated from *Erythrina abyssinica* and has not been published

2-(3,4-Dihydroxyphenyl)-3,5,7-trihydroxy-4H-1-benzo-pyran-4-one,3,3′,4′,5,6-pentahydroxyflavone (quercetin) (HPLC grade, ≥ 95%), N-tert-butyldimethylsilyl-N-methyltrifluoroacetamide (MTBSTFA) (≥ 99.0%), chloroform (ACS reagent, ≥ 99.8), dioxane (ACS reagent, ≥ 99.5%), acetonitrile (ACS reagent, ≥ 99.5%), and dimethyl sulfoxide (DMSO) (reagent grade, 99.5%) were all purchased from Sigma-Aldrich. Methanol (reagent grade, ≥ 99.4%) was purchased from Rochelle Chemicals. Disposable plastic syringes were used. RS Pro clear polypropylene (PP) filament was purchased from RS Components. All the reagents and materials were used as received. Initially, quercetin was used as a model compound to develop and optimize the silylation process. Thereafter, other flavonoids (TED 13, and ZTF 1016) previously isolated and characterized from plant extracts (see Fig. 1) were considered [33, 34].

### Derivatization

MTBSTFA was chosen for its stability, and it was used for all derivatization reactions in this study. All the flavonoids were derivatized and analyzed like quercetin unless stated otherwise.

In preparation of the batch samples, quercetin (0.5 mg/mL) was dissolved in a mixture of acetonitrile and dioxane (1:1, v/v). Thereafter, a 100 µL aliquot was pipetted into a dry GC vial with a 200-µL glass insert, and a 100 µL of MTBSTFA was subsequently added. The vial was sealed and homogenized using a vortex mixer for 1–2 min at room temperature and atmospheric pressure. The reaction mixture was subsequently analyzed using several analytical techniques. This approach was necessary because conventionally, derivatization reactions are performed in batch, and thus allowed for the validation of the newly developed flow method presented here.

**Fig. 2:**
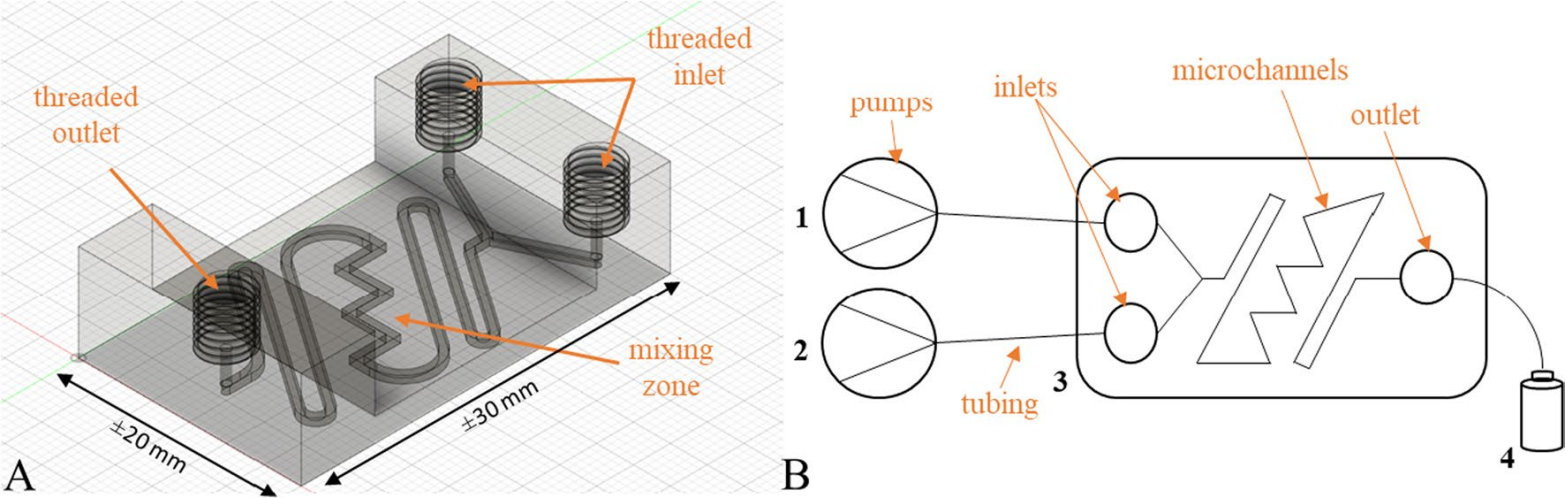
A CAD model of the microfluidic device (**A**) and a schematic diagram of the semi-automated continuous flow system (**B**) used for the derivatization of flavonoids. The system consists of syringe pumps (1 and 2), a microfluidic device (3), and sample collection (4)

A unique approach was used to translate the batch method to flow (see details in the supplementary information). A microfluidic device was manufactured and connected to the flow system, as shown in Fig. 2. The syringe pumps (automated fluid delivery system) (Fig. 2B) were connected to the Arduino board and controlled by a computer using the Poseidon pumps controller program [35]. The flavonoid solution and the MTBSTFA were introduced to the microfluidic device (Fig. 2A) at a combined flow rate of 200 µL/min. The effluent was collected in a GC vial for analysis.

### 3D printing of microfluidic devices

Initially, two different microfluidic devices were designed (Fig. 2 and S1). During preliminary investigations, it was observed that the derivatization reaction occurs more rapidly in the flow device and consequently the device and channel sizes were reduced, in an effort to reduce the residence (reaction) time. The final device (Fig. 2) was 30 × 20 mm (*l* × *b*), and had a channel size of 0.8 mm (ID). It is worth noting that this does not account for the PP shrinkage (≈ 20%), which is very common in 3D printing [32]. The mixing zone with 90° angles was moved to the middle section of the device to ensure improved mixing at constricted sizes [36]; this was necessary to promote diffusional mixing in laminar flow followed by rigorous mixing as reported by Ward and Fan (2015) that chaotic/rigorous mixing enhances mixing which results in faster reactions in microfluidics [37]. The modified device had a residence time of ≈ 3 min.

Digital models of the microfluidic devices were created using Autodesk® Fusion 360, an open-source computer-aided design (CAD) software program. The generated.stl file was further converted to a.gcode file using Prusa slicer. The.gcode file contains printing instructions such as the model dimensions, printing speed, bed, and nozzle temperatures [38]. The generated.gcode file was then transferred to the Prusa i3 MK3S + 3D printer (Prusa Research, Czech Republic).

The microfluidic devices were fabricated using a transparent polypropylene (PP) filament, a generally chemically resistant thermoplastic filament that also allows the reaction to be followed visually. The print bed (with clear packing tape) and nozzle (0.4 mm) temperatures were set at 85 °C and 230 °C, respectively. This was necessary to reduce stringing, warping, and shrinkage while also improving bed adhesion. To ensure leakproof devices, the layer height was set at 0.15 mm at 100% print speed, and the infill density and extruder flow were kept at 100% and 105%, respectively.

### Sample characterization

All the samples (flavonoids and derivatized flavonoids) were analyzed using a Shimadzu FTIR (QATR-S model) (Shimadzu, Japan). The background was blanked/zeroed by scanning before analyzing the samples. The spectrum was measured between 4000 and 400 cm^−1^ at a resolution of 16 cm^−1^, and 32 scans were recorded. The flavonoids were dissolved in an acetonitrile and dioxane mixture (1:1, v/v) prior to analysis.

GC–MS analysis was performed using a Shimadzu Nexis-2030 GC fitted with an AOC-20i auto-injector, coupled to GCMS-TQ8050 NX triple quadrupole MS (Shimadzu, Japan). A 30 m × 0.25 mm × 0.25 µm Restek Rxi-5HT high-temperature fused silica column (maximum column temperature of 400 °C) was used together with a 1.0 m × 0.25 mm Restek RxiGuard column. The oven temperature was initially held at 60 °C for 1 min, ramped to 320 °C at 20 °C/min, and finally ramped to 350 °C at 15 °C/min where it was held for 10 min. Ultrahigh-purity helium gas at 1.2 mL/min was used as a carrier gas. The inlet temperature was held at 280 °C throughout the analysis. A 20:1 split ratio with a 1-µL injection volume was used. The GC–MS interface was set at 250 °C and the ion source (electron ionization) at 200 °C. The EI mass spectra were acquired in the range of m/z 50–900. A Q3 scan mode with a scan speed of 3333 amu/sec was used for data acquisition. The data was processed using Shimadzu’s GCMSPRUN software. The samples were analyzed in full scan mode, and thus, no specific ions were isolated for the second scan.

The prepared samples were analyzed with a Synapt G2 HDMS (Waters Incorporated, MA, USA) equipped with electrospray ionization (ESI). The samples were analyzed in the high-resolution mode. Flow injection analysis was used and 5 µL was injected into the ionization source at 10 µL/min. The source and desolvation temperatures were set at 80 °C and 150 °C, respectively. The positive and negative ions were collected at + 4 kV and − 2.5 kV capillary voltages, respectively. The data were acquired and processed using MassLynx™ (version 4.1) software (Waters Incorporated, MA, USA).

## Results and discussion

### Quercetin

**Fig. 3:**
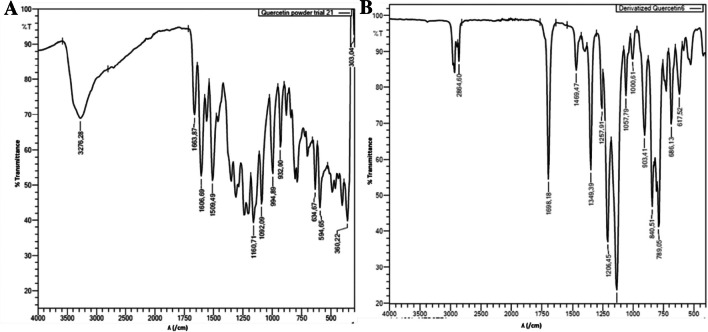
Fourier transform infrared spectrum of underivatized (**A**) and derivatized (**B**) quercetin. Acetonitrile and dioxane (1:1) was used as solvent

Quercetin contains five hydroxyl functional groups, and it is well-known that the peak characteristic of hydroxyl groups appears at 2400–3400 cm^−1^ on the FTIR spectrum. The peak is very broad in shape and is mainly due to the oxygen-hydrogen stretch. When the functional group interacts in hydrogen bonding, the peak tends to appear specifically at 3200–3500 cm^−1^ on the spectrum. A broad and intense peak was observed at 3276.28 cm^−1^ for underivatized quercetin, as expected (Fig. 3A). This peak was of utmost importance in this study because the hydroxyls are the target functional group for derivatization [4].

Derivatization (silylation) is characterized by replacing the acidic proton of the hydroxyl, thiol, and amine functional groups with a silyl group. However, this study mainly focused on the hydroxyl group, since the studied flavonoids do not contain amine or thiol groups. Upon derivatization of the quercetin sample, the previously observed OH-stretch peak in the underivatized sample disappeared, inferring that derivatization was successful (Fig. 3B). The observed peak at 2858.89 cm^−1^ is characteristic of a strong to C-H (sp, sp^2^, and sp^3^ hybridized) stretch and is usually overlapped by the hydroxyl group when present, as shown in Fig. 3B. Similar results were observed for samples derivatized in batch (not shown). For method validation, all the results for the samples prepared using the newly developed flow method were compared with the samples prepared in conventional manual batch method.

As a highly polar compound, quercetin has low volatility, making it difficult to analyze using GC. Nonetheless, an underivatized quercetin sample was analyzed in GC to confirm the theory. As expected, only the solvent peaks were observed on the chromatogram. Subsequently, quercetin was derivatized both in batch and in flow. Upon derivatization in batch and flow, a very distinct but low-intensity peak was observed at 21.952 min in the total ion chromatogram (TIC) (Fig. S2). According to the GC oven program, the oven temperature was 350 °C at this time. To ensure that the peak belonged to the derivatization product, sample blanks were also analyzed, and only the solvent and reagent peaks were observed.

Ions with m/z ratios of 530, 644, 758, and 872 were observed at 21.952 min (Fig. S3), and correspond to quercetin that has been derivatized at least two, three, four, and five times according to their respective predicted molecular formulas (C_27_H_38_O_7_Si_2_, C_33_H_52_O_7_Si_3_, C_39_H_66_O_7_Si_4_, C_45_H_80_O_7_Si_5_). It is important to note that electron impact ionization (EI) was used, and thus the observed ions could represent fragments of a single compound, and not necessarily a molecular ion. Therefore, the observed chromatographic peak may represent a single derivatized compound which fragmented upon ionization.

**Fig. 4:**
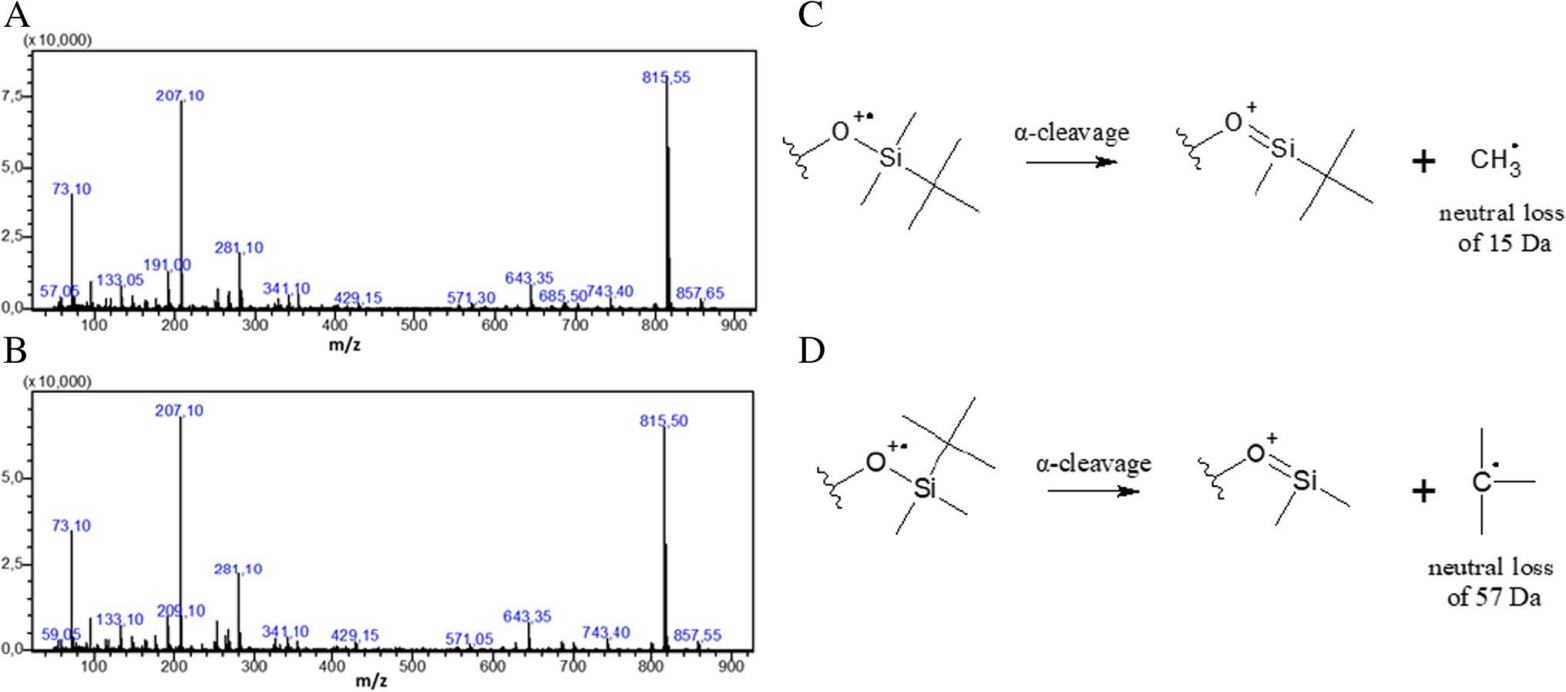
**A**–**D** EI mass spectrum of quercetin derivatized in batch (top) and flow (bottom) using MTBSTFA

Figure 4 shows the mass spectra obtained for this chromatographic peak (at 21.952 min) of quercetin derivatized both in batch and in flow. Notably, similar results were obtained. The molecular mass for quercetin that has been derivatized at least five times (with a predicted molecular formula of C_45_H_80_O_7_Si_5_) has an expected monoisotopic mass of 872.4752 Da. However, the observed peaks with m/z of 857 and 8515 were most likely due to α-cleavage, resulting in the loss of a methyl (Fig. 4C) and t-butyl (Fig. 4D) radical, respectively. The loss of a methyl or t-butyl group for other MTBSTFA-derivatized phenolic compounds has been reported [39]. The relative intensity of the peak at m/z 815 (relative to the peak at m/z 857) can be justified by the fact that the t-butyl radical is more stable than the methyl radical. The GC–MS results complement the FTIR data, and support the hypothesis that quercetin was successfully derivatized. The peak at m/z 73.10 was tentatively identified as a trimethylsilyl (TMS) radical/fragment and may be due to column bleed. The other peaks with m/z ratios of 191.00, 207.10, and 281.10 could not be assigned at this point; however, similar peaks were also observed in the Human Metabolome Database where quercetin was derivatized using TMS and subsequently analyzed using GC–MS [40].

**Fig. 5:**
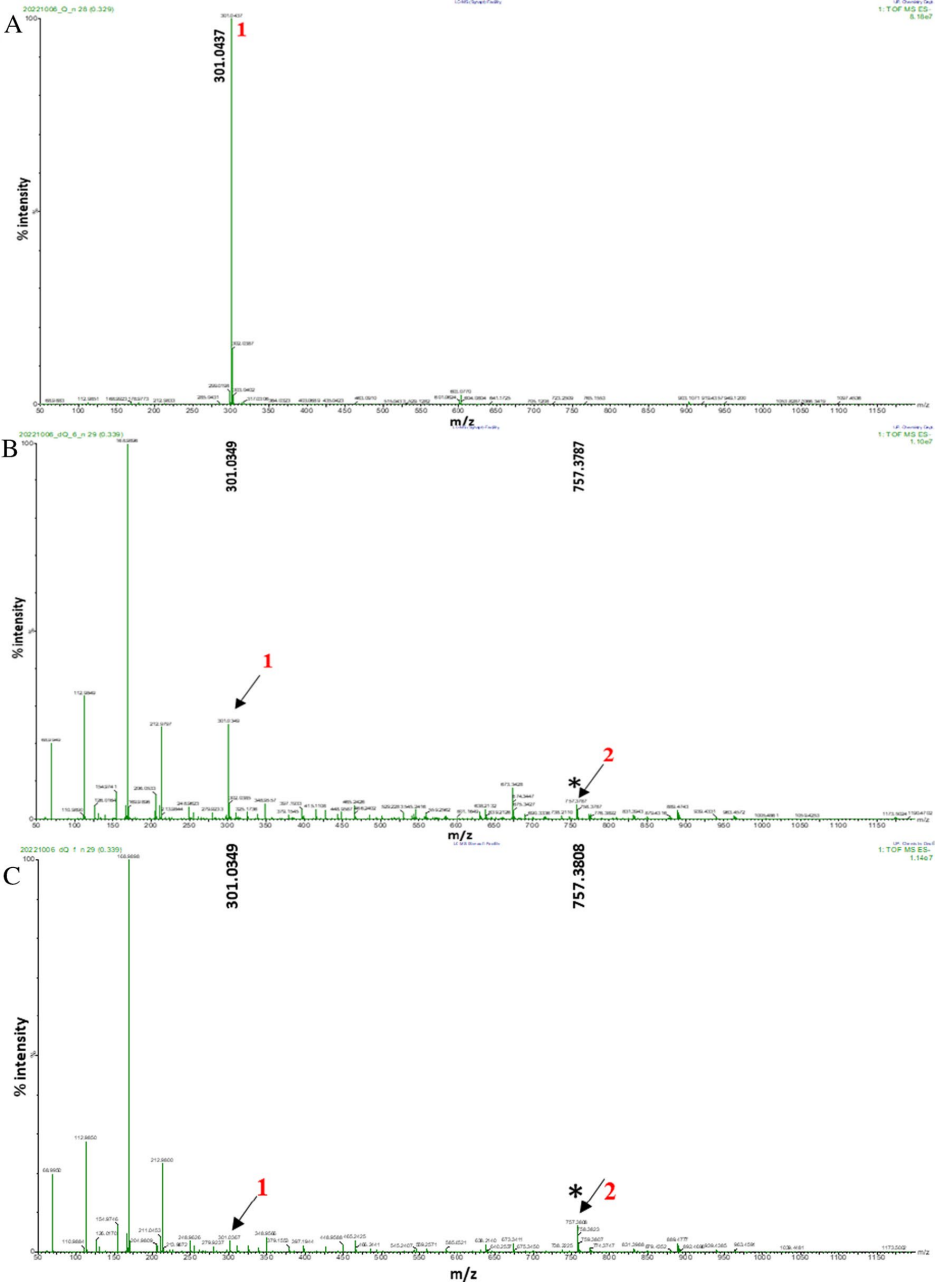
Negative ion HR-MS spectrum of targeted species obtained from analysis of quercetin samples. **A** The underivatized quercetin. **B** Quercetin derivatized in batch. **C** Quercetin derivatized in flow. The asterisk symbol indicates targeted (derivatized) peaks

Unlike in GC–MS, a soft ionization technique (i.e., ESI) was used for HR-MS analysis. In ESI, a compound either gains or loses a proton, producing quasi-molecular ions, [M + H]^+^ and [M–H]^–^, respectively. Quercetin was prepared and derivatized in batch and flow. The samples were analyzed in both negative (ESI −) and positive (ESI +) ionization modes and the results are shown in Fig. 5 and S4, respectively.

In Fig. 5, peak 1 represents the quasi-molecular ion of underivatized quercetin (predicted molecular formula C_15_H_9_O_7_) with an accurate mass of 301.0437 Da and a relative error of 10.1 mDa. Upon derivatization, peak 2 was observed both in the batch and flow samples (Fig. 5B and C) and represents the quasi-molecular ion (with an accurate mass of 757.3787 Da and 757.3808 Da) of quercetin that has been derivatized four times with a mass error of 1.1 mDa and − 0.2 mDa for batch and flow, respectively. Notably, the underivatized quercetin (peak 1) was observed in both derivatized samples, indicating incomplete derivatization. An average of 25 mass spectra was combined and used to get the relative areas of the identified peaks to perform a semi-quantitative analysis. For the batch and flow methods, the ratio of underivatized quercetin to derivatized quercetin was 9.08:1 and 0.366:1, respectively. This indicates that the flow system was 25 times more efficient than the batch system. Furthermore, two and five times derivatized quercetin (peaks 1 and 2) with m/z of 417.2466 and 873.4834, respectively, were observed in the positive ion mass spectrum (see Fig. S4).

### TED 13

The TED 13 sample was prepared, derivatized, and analyzed similar to quercetin. Unexpectedly, the typical hydroxyl characteristic peak (3200–3500 cm^−1^) was not observed for this sample. The FTIR spectra for both the derivatized and underivatized TED 13 were similar (Fig. S5), except for a peak observed at 2252.80 cm^−1^ exclusively for the derivatized sample. The peak is characteristic of a nitrile group, and was thus associated with the acetonitrile used to dissolve TED 13.

GC–MS analysis revealed a single small chromatographic peak at 17.375 min for the derivatized TED 13 (Fig. S6). The mass spectrum of this chromatographic peak for both the batch and flow samples (Fig. S7) did not contain any of the targeted peaks (m/z ratios of 388, 502, and 616). However, the peak with m/z of 559.35 corresponds to a loss of a t-butyl group (57 Da) from TED 13 that was derivatized three times, as reported in another study [39]. Similar low molecular weight ions to those observed for quercetin were observed in both batch and flow samples.

Results obtained from HR-MS analysis of TED 13 are shown in Fig. S8 and S9. For the negative ion spectra (Fig. S8), peak 1 represents the quasi-molecular ion of underivatized TED 13 (with a predicted molecular formula of C_14_H_9_O_6_) with an accurate mass of 273.0418 Da and a relative error of 1.8 mDa. Upon derivatization, peaks 2 and 3 were observed both in the batch and in flow samples (Fig. S8B and C); these peaks represent the quasi-molecular ions (m/z of 387.1264 and 501.2128) of TED 13 that has been derivatized once and twice according to their respective predicted molecular formulas (C_20_H_23_O_6_Si and C_26_H_37_O_6_Si_2_). Interestingly, the underivatized TED 13 (peak 1) was only observed in the batch derivatized sample, inferring that TED 13 was completely derivatized in flow. Therefore, this is complementary to the observations made for quercetin where the developed flow method was more efficient than the conventional batch method.

Similar results were observed in the positive mode (Fig. S9). Notably, peak 2 with a m/z of 617.3161 represents TED 13 that has been derivatized three times according to the predicted formula (C_32_H_52_O_6_Si_3_) and was observed both in batch and flow. Further, peak 1, which represents the underivatized TED 13 with predicted molecular formula (C_14_H_9_O_6_), was not observed in either the batch or flow derivatized samples, inferring complete derivatization.

### ZTF 1016

ZTF 1016 was recently isolated by our collaborators. The molecular structure (Fig. 1) was provisionally elucidated and provided to us with the sample. The sample was prepared and analyzed similarly to the other compounds. FTIR results showed a very small but broad peak observed at 3500 cm^−1^ (Fig. S10) for the underivatized ZTF 1016, which is characteristic of hydrogen-bonded hydroxyl functional groups. The intensity of this peak decreased significantly for the derivatized ZTF 1016 (Fig. S10). Similar results were observed for the batch and flow samples.

The GC–MS chromatogram showed a distinct peak at 17.220 min for both the batch and flow samples (Fig. S11). The mass spectrum for the observed peak on the chromatogram was obtained, and a distinct ion with m/z of 363 was observed (Fig. S12); however, it could not be attributed to any of the expected ions for the proposed structure. The observations were further investigated using HR-MS to provide valuable information about the compound’s identity and whether it was derivatized or not.

The ZTF 1016 samples were analyzed both in the ESI-negative and ESI-positive ionization modes. For the underivatized sample (Fig. S13) analyzed in positive mode, two peaks with m/z 379.1202 and 401.1002 were identified (labelled as peaks 1 and 2, respectively). The two peaks were attributed to the quasi-molecular ion, [M + H]^+^, and sodium adduct, [M + Na]^+^, respectively. Another peak with m/z 779.2098 (peak 3) was observed, corresponding to a sodium adduct of the dimer [2M + Na]^+^. Importantly, the observed masses do not correspond to the proposed structure of the flavonoid (Fig. 1). However, the results can be correlated to the GC–MS data, since the molecular mass would be 378 Da and thus the ion observed at a m/z of 363 (Fig. S12) corresponds to the loss of a methyl group (15 Da).

In the derivatized samples (batch and flow), the underivatized flavonoid peaks (1 and 2) were still observed. However, new peaks corresponding to the expected monoisotopic mass for peak 1 that was silylated once (493.2097 m/z) and four times (835.4821 m/z) were also observed (labelled as peaks 4 and 5, respectively). Elemental compositions were assigned to these peaks using the MassLynx software. Based on this information, the newly proposed molecular formula for this compound is C_15_H_22_O_11_, resulting in a mass error of − 1.1 mDa for the observed [M + H]^+^ ion. This confirms that derivatization was achieved, but it was incomplete since the underivatized compound was still present. Furthermore, in batch, it was observed that the compound was derivatized only once, while in flow it was derivatized once and four times (i.e., peak 5 was not observed for the sample derivatized in batch). The peaks observed in the negative mode did not correspond to the peaks observed in positive mode and will be further investigated in future. However, the positive ion mode data infers that the flow method was more efficient than the batch method, as observed with TED 13 and quercetin samples.

Importantly, the results for ZTF 1016 illustrate how derivatization can provide an additional dimension of information to elucidate the structure of an unknown compound. This is a novel approach with significant potential in the field of natural product analysis. HR-MS provided an accurate mass that could be used to predict the molecular formula of the flavonoid (first column in Table 2). However, the accurate masses of the derivatized species provide new information. By cross-checking the predicted formulas for ions with m/z 379.1202 and 493.2097 (observed for the batch method), four possible molecular formulas (differing with the required C_6_H_14_Si group that indicates silylation) were obtained (second column in Table 2). The flow method provides additional information since the flavonoid was derivatized one and four times, indicating that it has to have at least four hydroxyl groups (thus four O-atoms). Furthermore, the predicted formulas for the derivatized species had to contain one and four Si atoms, respectively. By cross-checking the predicted formulas for the observed ions (Table 1), the list of likely molecular formulas was narrowed down to a single possible formula (third column in Table 2). This would not have been possible if derivatization was done with the traditional batch method, since the reaction was less efficient and only one hydroxyl group was derivatized. Based on these results, further analyses to elucidate the structure of ZTF 1016 will be done in future.

**Table 2:** Predicted molecular formulas for the underivatized and MTBSTFA derivatized ZTF 1016 analyzed using HR-MS in positive (ESI +) mode

Underivatized [M + H]^+^ ion (m/z 379.1179)	Derivatized once (m/z 493.2097)	Derivatized four times (m/z 835.4821)
C_22_H_19_O_6_^a^	C_28_H_33_O_6_Si^a^	C_50_H_75_O_3_Si_4_
C_29_H_15_O^a^	C_35_H_29_OSi^a^	C_43_H_79_O_8_Si_4_
C_11_H_23_O_14_^a^	C_17_H_37_O_14_Si^a^	C_36_H_83_O_13_Si_4_
C_15_H_23_O_11_^a,b^	C_21_H_37_O_11_Si^a,b^	C_39_H_79_O_11_Si_4_^b^
C_18_H_19_O_9_	C_32_H_33_O_3_Si	C_54_H_75_ Si_4_

^a^Possible formulas if only first derivatization is taken into account

^b^Possible formulas if information from derivatization in flow is taken into account

## Conclusion

The aim of this study was to automate the silylation of flavonoids using 3D printed microfluidics to expand the applicability of GC in flavonoid analysis, since it is a simpler and greener chromatographic method compared to traditional HPLC. Flavonoids were successfully derivatized in flow at room temperature as validated by critical analysis of the reaction products obtained in batch and flow reactions. FTIR was used to confirm that the hydroxyl groups were no longer present after the derivatization reaction. GC–MS was used to confirm the physicochemical transformation making the flavonoids amenable to GC analysis, and HR-MS was used to confirm the elemental composition of the reaction products. Importantly, based on the relative abundance of derivatized and underivatized compounds observed with HR-MS, higher degrees of silylation and in some cases complete reactions (with no underivatized compounds detected) were observed when comparing the developed flow method to the conventional batch method. This study has also shown for the first time that derivatization can provide additional information related to structure elucidation of flavonoids, and a new molecular formula for one of the flavonoids was deduced. Therefore, an alternative, semi-automated, and sustainable natural product derivatization method based on 3D printed continuous flow chemistry has been demonstrated and proved more efficient compared to the conventional manual batch method.

### Supplementary Information

Below is the link to the electronic supplementary material.

Supplementary file1 (DOCX 1220 KB)
