# Supplementary material for: Automated silylation of flavonoids using 3D printed microfluidics prior to chromatographic analysis: system development
Source: Anal Bioanal Chem. 2023 Oct 7;415(29-30):7151–60. doi: 10.1007/s00216-023-04981-4 (PMC10684624; doi:10.1007/s00216-023-04981-4)
Supplement: Supplementary file 1 — Supplementary file1 (DOCX 1220 KB) [file 216_2023_4981_MOESM1_ESM.docx]

Automated Silylation of Flavonoids using 3D Printed Microfluidics prior to Chromatographic Analysis: System Development

Thabang Bernette Ncongwane^1^, Derek Tantoh Ndinteh^1^, Elize Smit^1*^

^1^ Center for Natural Products Research, Department of Chemical Sciences, University of Johannesburg, PO Box 524, Auckland Park, Johannesburg, South Africa

* Corresponding author: [esmit@uj.ac.za](mailto:esmit@uj.ac.za)

**Method development: Translation of batch to flow**

In this study, the translation of batch reactions into flow reactions was implemented using a two-step approach that provided for a smoother transition. The batch reactions were first translated into flow using continually stirred tank reactors (CSTRs). CSTRs originally developed by Neumaier et al. and later modified by du Preez et al. were adopted [1, 2]. These reactors can be classified as a “batch in flow” system since the tank volume is relatively large (≈ 1.2 mL), representative of in-vial reactions. However, unlike batch reactions, CTSRs consists of a constant flow in and out of the tank. The prepared 0.5 mg/mL quercetin sample was added into a 5 mL syringe, and a 1 mL syringe was filled with MTBSTFA, of which both were respectively fitted to the open-source syringe pump system originally developed by the Pachter group [3]. The syringe pumps were connected to the CSTRs via threaded flangeless nuts and Teflon^TM^ tubing. During trial reactions and optimizations, the total flow rate was varied between 100 - 500 µL/min, and 200 µL/min was found to be an optimal flow rate. Both the sample and the reagent (MTBSTFA) in the syringes were delivered at 100 µL/min each. The CSTRs had a residence time of ± 10 minutes, and three fractions were collected and analyzed similar to the samples prepared in batch. Once the flow rates were optimized for the CSTRs, the reactions were further translated to a channel-based device.

Different designs for the flow channel-based devices were considered. Factors such as print quality, channel internal diameter (ID), device size (which influences residence time) and connection ports need to be considered for a successful working microfluidic device. For this application, the first design (Fig. S1) involved a larger device of 40 × 40 mm (*l × b*), with ± 1 mm ID channels, the threaded connection ports were also designed to fit commercial flangeless nuts (¼ - 28 UNF), as shown in Fig. S1. The device offered a residence time of ≈ 5 minutes at a 200 µL/min combined flow rate. It was observed that the derivatization reaction occurs more rapidly in the flow device compared to the CSTRs and the batch reactions, indicating that the residence (reaction) time could be reduced. Hence, an even smaller device was designed, where both device and channel sizes were reduced (Fig 2).


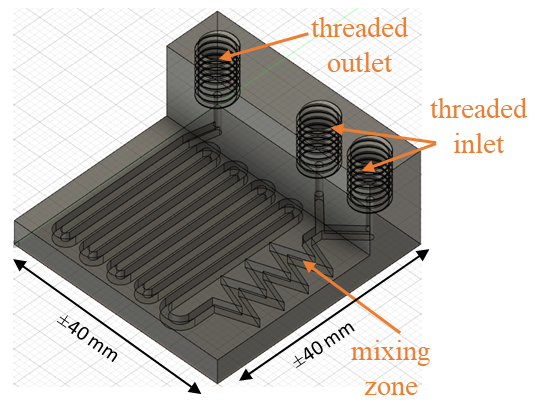


Fig. S1 CAD model of the channel device (microfluidics) with 40 × 40 mm dimensions. The channels are ±1 mm in internal diameter (ID), and the enclosed device has two threaded inlets and one outlet for tube connection via flangeless nuts.

**Results: Supplementary Chromatograms and Spectra**


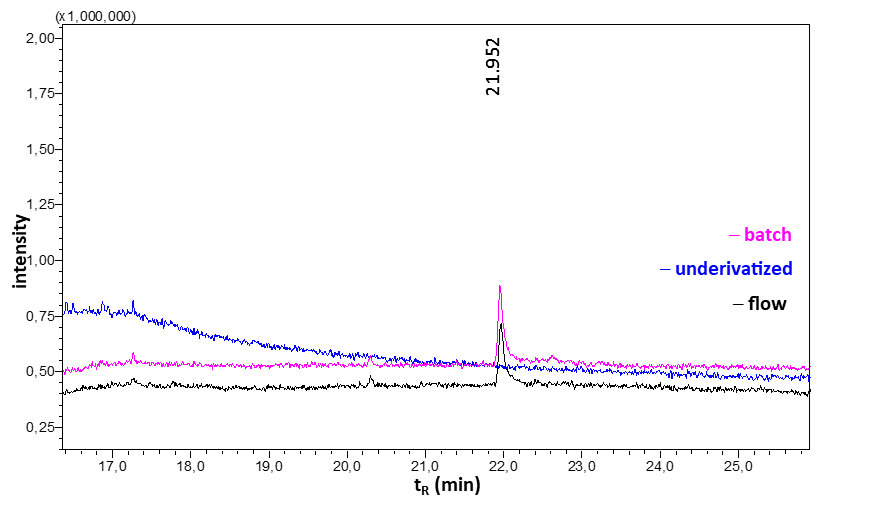


**Fig. S2** GC-MS total ion chromatogram (TIC) of underivatized quercetin (blue), quercetin derivatized in batch (magenta), and in flow (black) using MTBSTFA.


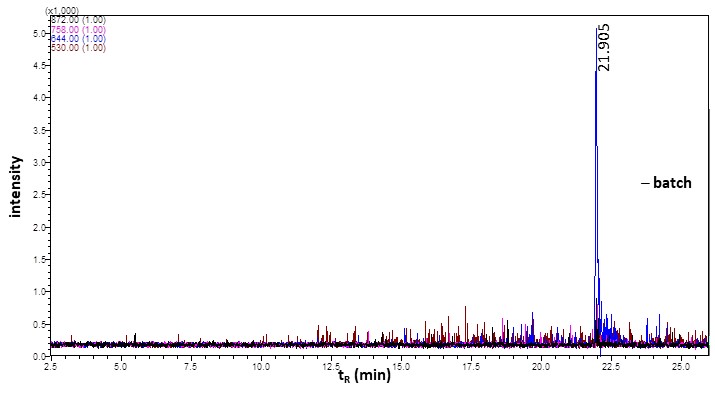

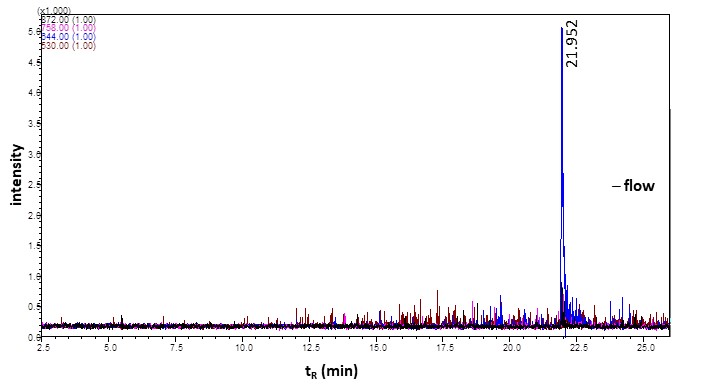


**Fig. S3** GC-MS extracted ion chromatograms (EIC) of ions with m/z of 530, 644, 758, and 872 for a quercetin sample derivatized in batch and flow. These ions correspond to the expected masses of quercetin that has been derivatized up to five times.


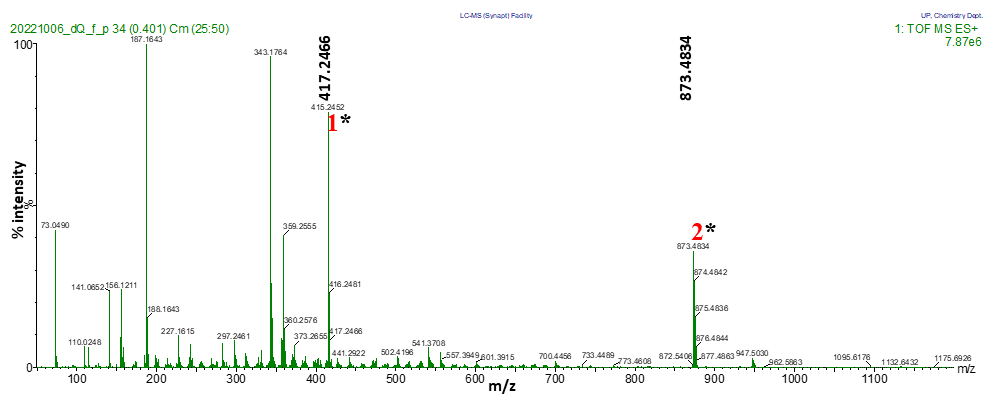


Fig. S4 Positive ion ESI HR-MS spectrum of targeted species obtained from analysis of quercetin derivatized in flow. Similar results were observed for quercetin derivatized in batch (not shown). *Targeted (derivatized) peaks.


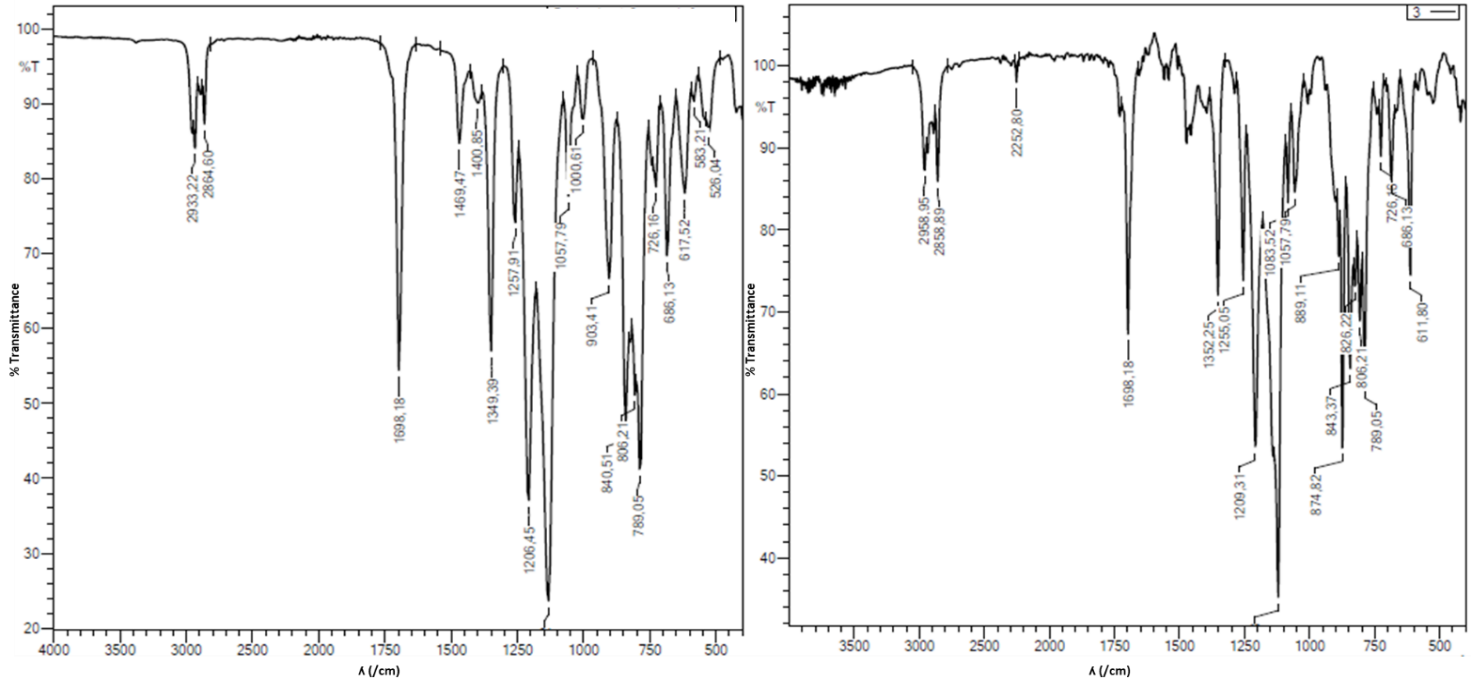


**Fig. S5** Infrared spectrum of TED 13 dissolved in 1:1 (v/v) acetonitrile and dioxane mixture (left) and derivatized in flow using MTBSTFA (right).


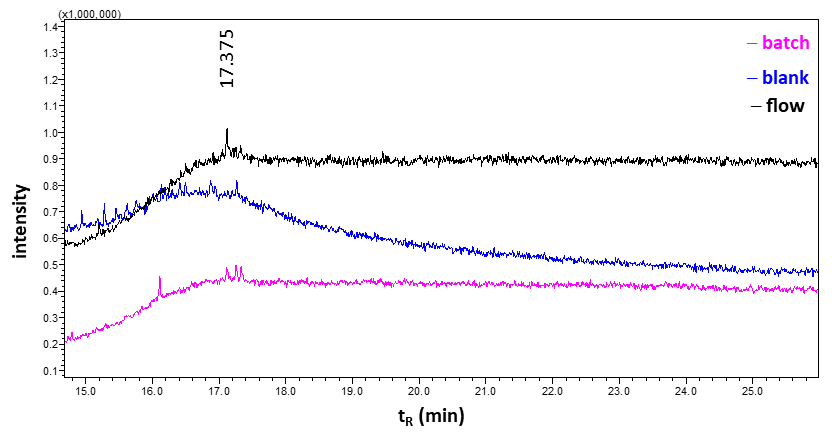

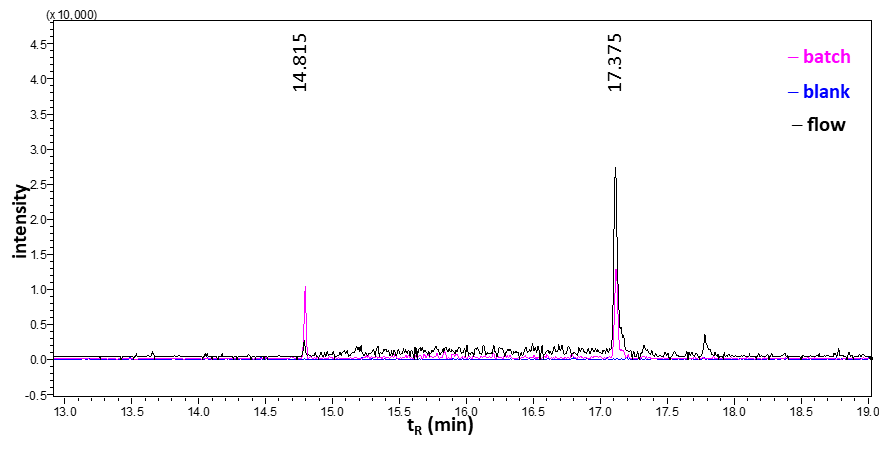


**Fig. S6** GC-MS total ion chromatogram (TIC, left) and extracted ion chromatogram (EIC, right) of the blank sample (blue), TED 13 derivatized in batch (magenta), and in flow (black) using MTBSTFA. The EIC represents a m/z of 559 which corresponds to the loss of a t-butyl group (M-57) from TED 13 that was derivatized 3 times (M = 616).


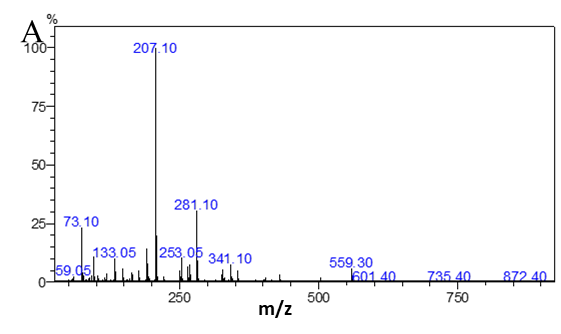


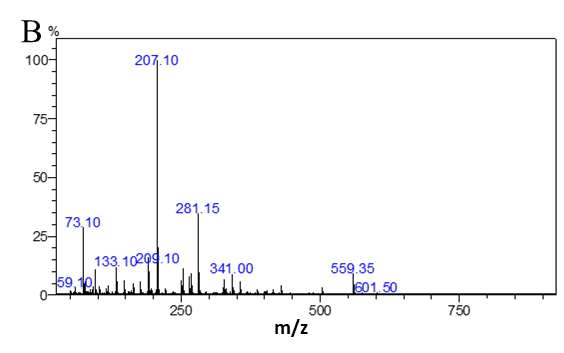


Fig. S7 EI-mass spectrum of TED 13 derivatized in batch (top) and flow (bottom) using MTBSTFA.


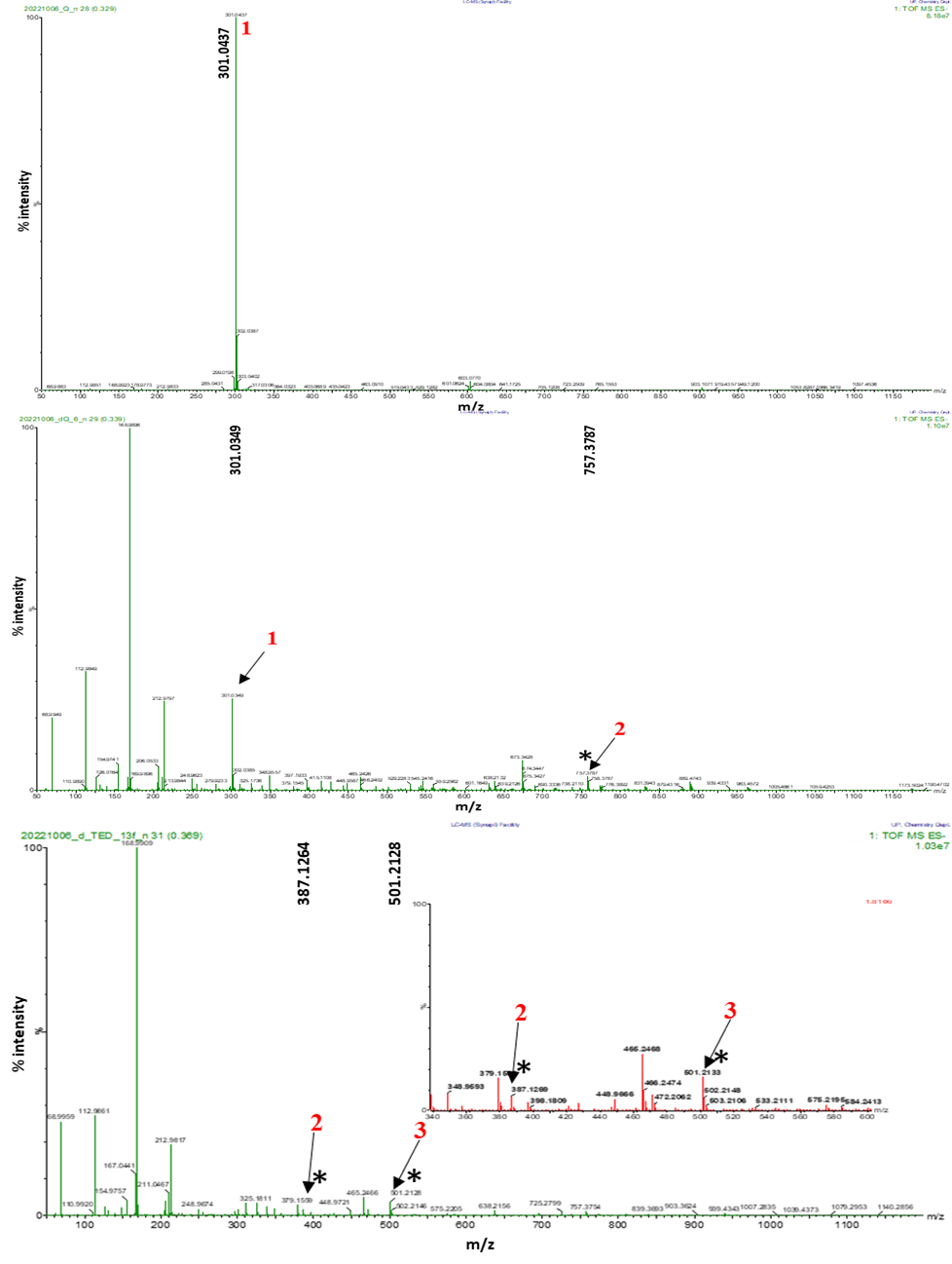


Fig. S8 Negative ion ESI HR-MS spectrum of targeted species obtained from analysis of TED 13 samples. (A) represents the underivatized TED 13, (B) represents TED 13 derivatized in batch and (C) TED 13 derivatized in flow. *Targeted (derivatized) peaks.


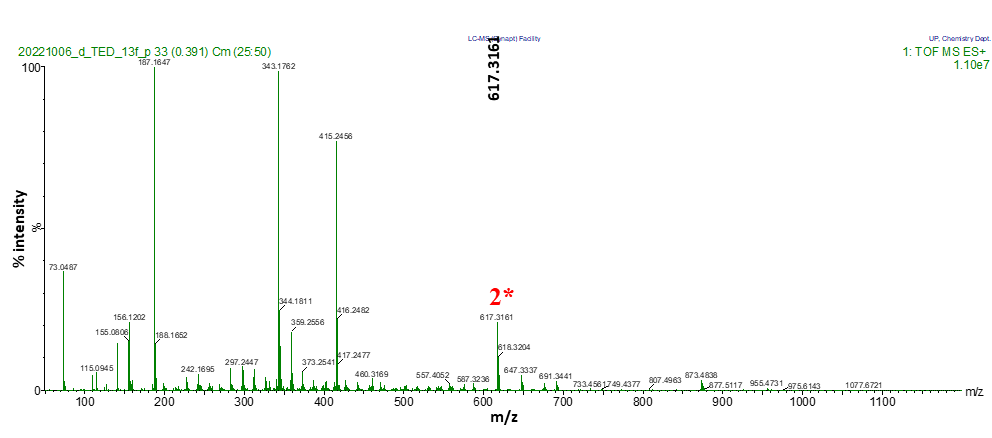


Fig. S9 Positive ion HR-MS spectrum of targeted species obtained from analysis of TED 13 derivatized in flow. *Target (derivatized) peaks.


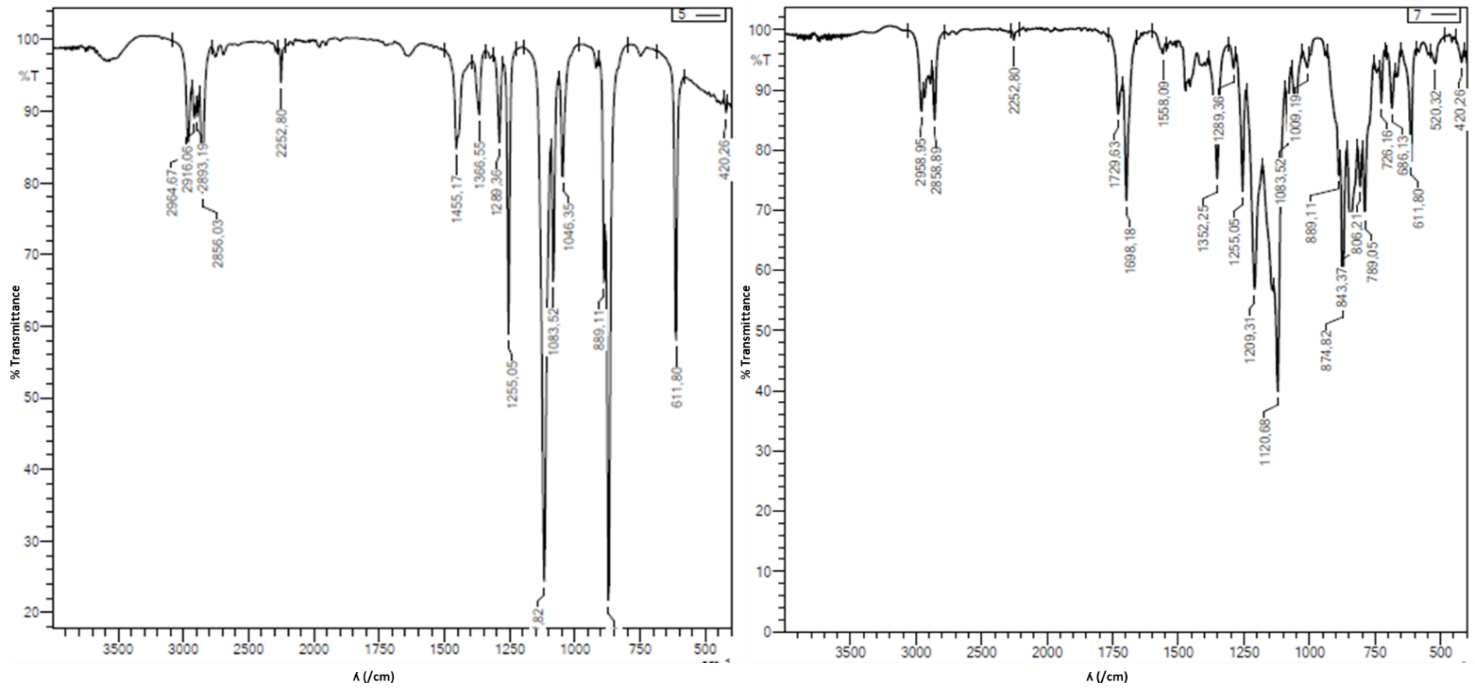


**Fig. S10** Infrared spectrum of ZTF 1016 dissolved in a 1:1 (v/v) acetonitrile and dioxane mixture (left) and derivatized in flow using MTBSTFA (right).


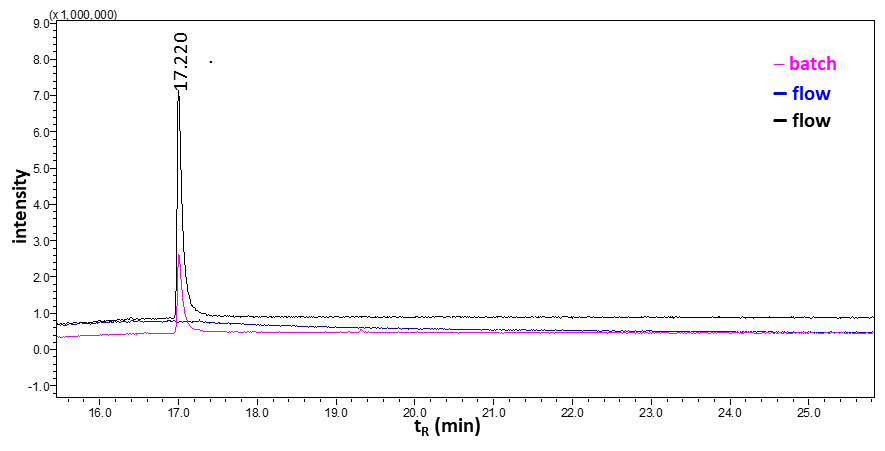


**Fig. S11** GC-MS total ion chromatogram (TIC) of ZTF 1016 derivatized in batch (magenta), and in flow (black) using MTBSTFA.


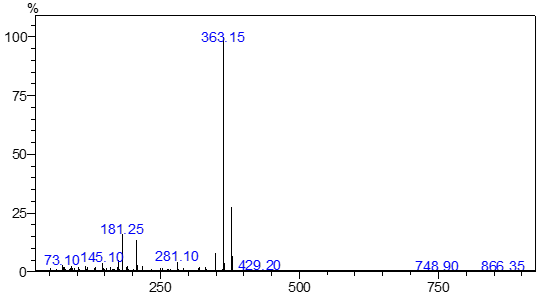

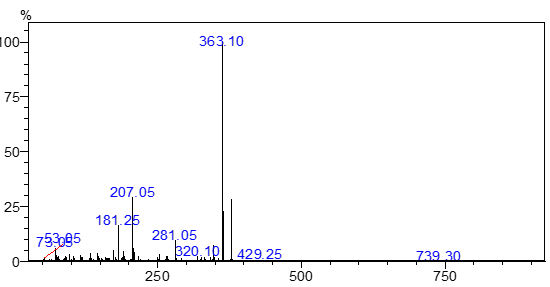


**Fig. (S12)** Mass spectra of chromatographic peak at 17.220 mins of ZTF 1016 derivatized in batch (top) and in flow (bottom) respectively, using MTBSTFA.


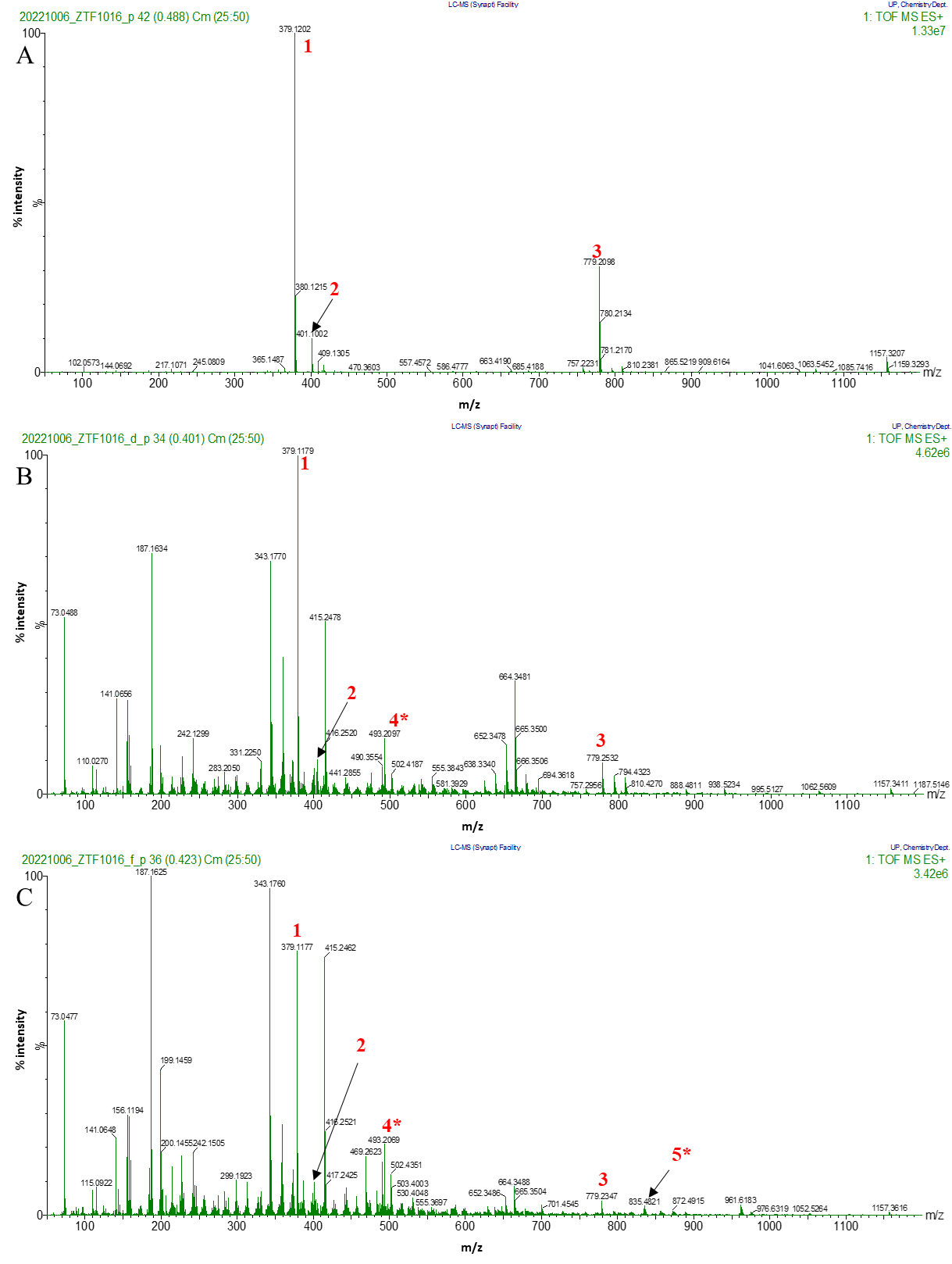


Fig. S13 Positive ion HR-MS spectrum of targeted species obtained from analysis of ZTF 1016 samples. (A) represents the underivatized ZTF 1016, where peak 1, 2 and 3 were identified as [M+H]^+^, [M+Na]^+^ and [2M+Na]^+^ respectively. (B) represents ZTF 1016 derivatized in batch and (C) represents ZTF 1016 derivatized in flow. *Target (derivatized) peaks.

**References**

1. Neumaier JM, Madani A, Klein T, Ziegler T (2019) Low-budget 3D-printed equipment for continuous flow reactions. Beilstein J Org Chem 15:558–566. https://doi.org/10.3762/bjoc.15.50

2. du Preez A, Meijboom R, Smit E (2022) Low-Cost 3D-Printed Reactionware for the Determination of Fatty Acid Content in Edible Oils using a Base-Catalyzed Transesterification Method in Continuous Flow. Food Anal Methods 15:1816–1825. https://doi.org/10.1007/S12161-022-02233-2/FIGURES/8

3. Booeshaghi AS, Beltrame E da V, Bannon D, Gehring J, Pachter L (2019) Principles of open source bioinstrumentation applied to the poseidon syringe pump system. Sci Rep 9:12385. https://doi.org/10.1038/s41598-019-48815-9
